# Preferred Mode of Therapy Among Patients in Rheumatoid Arthritis Saudi Database: A Cross-Sectional Study

**DOI:** 10.7759/cureus.41014

**Published:** 2023-06-27

**Authors:** Rahaf I Bukhari, Rasha Alamr, Ranin A Alsindi, Bayan F Hafiz, Aziza A Gadah, Nermeen A Awad, Mohamed Cheikh, Fatma Alshaiki, Suzan M Attar, Gamal Attia, Hani M Almoallim

**Affiliations:** 1 Department of Medicine, Faculty of Medicine, Umm Al-Qura University, Makkah, SAU; 2 Department of Medicine, Umm Al-Qura University, Makkah, SAU; 3 Department of Medicine, Dr. Soliman Fakeeh Hospital, Jeddah, SAU; 4 Department of Rheumatology, Dr. Soliman Fakeeh Hospital, Jeddah, SAU; 5 Department of Rheumatology, East Jeddah Hospital, Jeddah, SAU; 6 Department of Medicine, Faculty of Medicine/Rheumatology, King Abdulaziz University, Jeddah, SAU; 7 Department of Medicine, Faculty of Medicine/Rheumatology, Al-Azhar University, Cairo, EGY; 8 Department of Medicine, Faculty of Medicine/Rheumatology, Umm Al-Qura University, Makkah, SAU

**Keywords:** saudi arabia, mode of therapy, patients’ preference, drug administration, rheumatoid arthritis

## Abstract

Aims and objectives: Current knowledge of patients’ preferences for rheumatoid arthritis (RA) treatment is limited. Our goal was to determine the most favorable mode of therapy and the reasons behind choosing each route among RA patients in the Rheumatoid Arthritis Saudi Database (RASD).

Materials and methods: In this cross-sectional, nationwide, qualitative study, we conducted interviews with 308 RA patients to assess their preferred mode of therapy (oral, subcutaneous (SC) injection, or intravenous (IV) infusion) and to determine the reasons behind their choice. The determining factors behind patients’ preferred mode of therapy were evaluated using a 10-point allocation system (1 = least important, 10 = most important).

Results: We interviewed 308 RA patients (83.4% females, mean age, 48 years). Among all administration modes, the oral route was identified as the most preferred mode among our patients, with a percentage of 73.3%, followed by SC injection and IV infusion at 19.5% and 7.3%, respectively. Ease of drug administration was the most reported reason for patients who chose the oral route over the injection route (63.2%). Difficulty remembering to take the drug and finding it hard to swallow the pills were the highest-scored reasons for avoiding the oral route (24.9%).

Conclusion: Our study demonstrates and emphasizes the importance of shared decision-making between patients and their physicians. The oral route of therapy is, by far, the most preferred mode among our cohort of RA patients.

## Introduction

Rheumatoid arthritis (RA) is a chronic inflammatory autoimmune disease that progressively affects joints, bones, synovial tissues, and other structures. The severity of the disease can significantly impact patients’ quality of life, often resulting in difficulties performing basic daily activities. Therefore, RA treatment plans aim for disease remission or low disease activity to optimize patients’ health and improve their lifestyles.

Several types of treatments are available for RA, known as disease-modifying antirheumatic drugs (DMARDs). These are divided into three subtypes: conventional synthetic DMARDs (csDMARDs, e.g., methotrexate, hydroxychloroquine), biologic DMARDs (bDMARDs, e.g., tumor necrosis factor inhibitors (TNF)- α inhibitors, interleukin-6 receptor inhibitors, anti-CD20 monoclonal antibody (Rituximab), and a T-cell costimulation modulator), and targeted synthetic DMARDs (tsDMARDs, e.g., Janus kinase inhibitors). Each type of DMARDs has a different mode of action, use, and route of administration, which can vary from oral intake, subcutaneous (SC) injection to intravenous (IV) infusion. Patients’ preferences play a significant role in medication adherence, potentially leading to better disease outcomes and higher rates of remission.

This study was conducted to fill a gap in RA management in Saudi Arabia, gaps like delay in diagnosis [1], the prevalence of RA [2], and the comparative effectiveness of different drugs. Our aim is to understand our patients’ preferences and the reasons behind them. We believe this knowledge will assist rheumatologists in their shared decision-making process with their patients. Additionally, it will provide a foundation for policymakers to devise strategic plans for RA management in the country.

## Materials and methods

Subjects

This is a cross-sectional study that included 308 RA patients from five different hospitals located in the western and southern regions of Saudi Arabia, who actively participated in our questionnaire.

Objectives

The primary objective of the study was to identify the preferred mode of therapy among RA patients in Saudi Arabia. The secondary objectives were to investigate the factors and reasons influencing our patients’ choices.

Questionnaire design

All eligible participants were formally contacted by the principal investigator (PI) of the study at each center, followed by further contact by trained researchers and data collectors.

The designed questionnaire consisted of two sections. The first section pertained to demographic data, including the patient’s gender, age, marital status, place and type of residence, educational level, employment status, weight, height, and smoking status.

The second section of the questionnaire focused on each patient’s preferred mode of DMARDs administration and the factors contributing to that choice. These factors were framed as a scale composed of 23 questions, with each criterion ranging from “not important at all = 1” to “very important = 10”.

Subsequently, each patient was asked to choose their preferred mode of therapy, the reasons behind that choice, and the reasons for finding other modes of administration unfavorable.

Additionally, to effectively examine each patient’s choice and level of medication adherence, other questions were posed specifically about their ongoing medications, their knowledge about rheumatoid diseases, and their relationship with the treating physician (some of the results from this section are not presented in this manuscript).

Inclusion and exclusion criteria

The eligibility criteria included patients of both genders, aged 18 years or older, residing in Saudi Arabia, diagnosed with RA, and treated with at least one biologic therapy. Patients who refused to give informed consent or who did not meet the inclusion criteria were excluded.

Statistical analysis

As the prevalence of the preferred mode of therapy was not known, we used 50% prevalence, to calculate the sample size. Other factors considered were 95% confidence interval (CI) and 5% level of significance and the final sample size was calculated as 384. However, we were able to interview only 308 participants due to various reasons. Data from a total of 308 patients were entered, edited, and cleaned using Statistical Product and Service Solutions (SPSS) (IBM SPSS Statistics for Windows, Version 22.0, Armonk, NY). Categorical data was measured using percentages, and statistical significance was determined by applying the chi-square test of significance. A P-value less than 0.00 was considered to indicate a significant difference. The CI was set at 95%. Continuous data was measured using mean and standard deviation, and the student’s t-test was applied to identify significant differences.

## Results

Patient characteristics

A total of 308 patients were enrolled in the study. However, one patient did not have complete information. Hence, data from 307 patients were analyzed. The mean age of the patients was 48 years, with female dominance (83.4%). In terms of marital status, 75.9% were married, and 11.7% were single. Patients living in a city constituted 88.3% of the sample, while those living in a village made up 11.7%. More than half of our study sample resided in the western region of Saudi Arabia (60.3%). Furthermore, most patients had attained a university degree (49.5%), followed by patients with a primary school degree (18.6%). Further socio-demographic details are presented in Table 1.

**Table 1:** Demographic characteristics of RA patients (n = 307) RA: rheumatoid arthritis

Demographic characteristics	
Gender, n (%)	
Female	256 (83.4%)
Male	51 (16.6%)
Age, mean (SD)	48 (12.6)
Weight, mean (SD)	75 kg (16.7)
Height, mean (SD)	159 cm (8.9)
Body Mass Index (BMI), mean (SD)	29.33 (5.7)
Marital Status, n (%)	
Married	233 (75.9%)
Single	36 (11.7%)
Widowed	23 (7.5%)
Divorced	15 (4.9%)
Type of Residence, n (%)	
City	271 (88.3%)
Village	36 (11.7%)
Place of Residence, n (%)	
Central Region	16 (5.2%)
Eastern Region	4 (1.3%)
Northern Region	9 (2.9%)
Southern Region	93 (30.3%)
Western Region	185 (60.3%)
Educational Level, n (%)	
University Degree	152 (49.5%)
Primary School	57 (18.6%)
High School	55 (17.9%)
Literate/No Formal Education	4 (1.3%)
None	39 (12.7%)
Employment Status, n (%)	
Employed	93 (30.3%)
Unemployed	183 (59.6%)
Retired	31 (10.1%)
Smoking, n (%)	
Yes	35 (11.4%)
No	272 (88.6%)

Influential factors for selecting the most favorable mode of therapy

The factors influencing patients’ choices when selecting their preferred mode of therapy are shown in Table 2. Using a 10-point scale (1 = not important, 10 = very important), patients were asked to score 10 factors that influence their choice of the preferred mode of administration. Ease of handling and using the medication, being able to administer the treatment at home, and ease of recall were scored as the most important factors by 46.9%, 46.6%, and 45% of the patients, respectively. On the other hand, the disposal of needle syringes and the taste of medications were scored as the least important factors in selecting the most favorable mode of therapy, with percentages of 42.7%, and 38.8%, respectively. Further details are listed in Table 2.

**Table 2:** Factors influencing patients’ choice for their most preferred mode of administration using a 10-point allocation (1 = not important, 10 = very important) (n= 307)

Ease of handling and use of the medication										
Score	1	2	3	4	5	6	7	8	9	10
n	31	5	5	4	25	15	14	27	37	144
%	10.1%	1.6%	1.6%	1.3%	8.1%	4.9%	4.6%	8.8%	12.1%	46.9%
Ease of recall										
Score	1	2	3	4	5	6	7	8	9	10
n	29	6	5	6	30	13	22	25	33	138
%	9.4%	2%	1.6%	2%	9.8%	4.2%	7.2%	8.1%	10.7%	45%
Ease of obtaining the medications										
Score	1	2	3	4	5	6	7	8	9	10
n	21	3	4	6	18	18	17	41	42	137
%	6.8%	1%	1.3%	2%	5.9%	5.9%	5.5%	13.4%	13.7%	44.6%
Repetition of doses										
Score	1	2	3	4	5	6	7	8	9	10
n	64	5	9	12	19	16	24	18	30	110
%	20.8%	1.6%	2.9%	3.9%	6.2%	5.2%	7.8%	5.9%	9.8%	35.8%
No disposing of needle syringe										
Score	1	2	3	4	5	6	7	8	9	10
n	131	4	9	11	29	17	12	18	13	63
%	42.7%	1.3%	2.9%	3.6%	9.4%	5.5%	3.9%	5.9%	4.2%	20.5%
Ease of medication storage										
Score	1	2	3	4	5	6	7	8	9	10
n	78	2	3	6	14	8	18	24	25	129
%	25.4%	0.7%	1%	2%	4.6%	2.6%	5.9%	7.8%	8.1%	42%
Painless										
Score	1	2	3	4	5	6	7	8	9	10
n	106	10	12	5	30	16	17	23	21	67
%	34.5%	3.3%	3.9%	1.6%	9.8%	5.2%	5.5%	7.5%	6.8%	21.8%
Can be administrated at home										
Score	1	2	3	4	5	6	7	8	9	10
n	40	2	4	6	12	11	23	39	27	143
%	13%	0.7%	1.3%	2%	3.9%	3.6%	7.5%	12.7%	8.8%	46.6%
Ease of swallowing										
Score	1	2	3	4	5	6	7	8	9	10
n	88	9	12	13	23	24	17	26	18	77
%	28.7%	2.9%	3.9%	4.2%	7.5%	7.8%	5.5%	8.5%	5.9%	25.1%
Taste										
Score	1	2	3	4	5	6	7	8	9	10
n	119	19	16	7	21	17	13	24	13	58
%	38.8%	6.2%	5.2%	2.3%	6.8%	5.5%	4.2%	7.8%	4.2%	18.95

Treatment mode preferences

**Figure 1:**
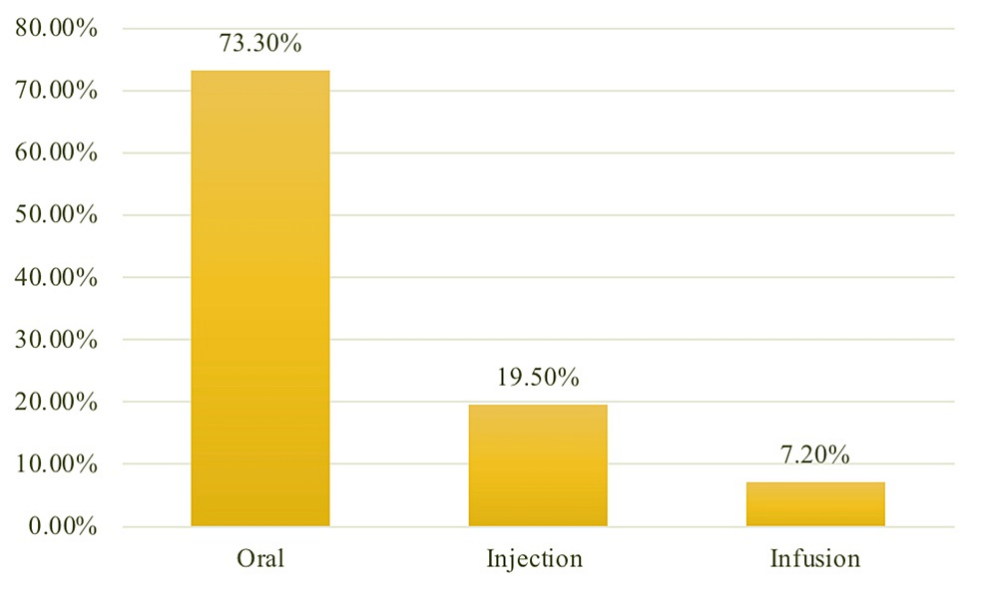
Most preferred mode of therapy among RA patients (n=307) RA: rheumatoid arthritis

Across all three treatment modes, oral administration was chosen by the majority of our patients as the most preferred mode of therapy, with a percentage of 73.3%, followed by SC injection (19.5%), and IV infusion (7.2%), as shown in Figure 1.

Reasons for treatment mode preferences

Table 3 presents the most common reasons for choosing and avoiding each mode of therapy. Patients who chose oral administration as their most preferred mode of treatment commonly cited multiple reasons for their preference, including ease and speed of administration, and portability of medications, with a percentage of 63.2%. The majority of patients who preferred the injection route also reported multiple reasons, such as ease and speed of administration, convenience of use, and frequency of medication doses, with a percentage of 19.2%. Only 7.2% of our patients chose IV infusion as their most preferred mode of therapy, with reasons including feeling comfortable with someone else administrating the medication, frequency of medication doses, and a feeling of safety, with a percentage of 6.2%.

Patients who did not prefer the oral mode reported multiple reasons, including difficulty in remembering to take the drug and difficulty swallowing the pills, with a percentage of 24.9%. Nearly all of our patients avoided the SC injection route, with the majority citing pain caused by needles and the frequency of the SC injections (71%) as the most common reasons for avoidance. Among patients who avoided IV infusions, inconvenience of the route and lengthy infusion times were cited as reasons, with a percentage of 29.3%. Further details are listed in Table 3.

**Table 3:** Most common reasons for choosing and avoiding oral administration, infusion, and injection route as the most preferred mode of therapy (n= 307)

Mode of therapy	n	%
Oral administration	225	73.3%
Reasons for choosing oral route:		
Ease of administration	32	10.4%
Speed of administration	1	0.3%
Portability	12	3.9%
Multiple reasons (Ease of administration, speed of administration, and portability of the medication)	194	63.2%
Reasons for avoiding oral route:	82	26.7%
Difficulty to remember taking the medications	5	1.6%
Multiple reasons (Difficulty to remember taking the medication, hard to swallow, and possible drug-drug interaction)	77	24.9%
Injection administration		
Reasons for choosing injection route:	60	19.5%
Ease of administration	1	0.3%
Multiple reasons (Ease and speed of administration, convenient to use, and frequency of medications doses)	59	19.2%
Reasons for avoiding injection route:		
Avoid because of pain caused by needles	13	4.2%
Multiple reasons (Avoid because of pain caused by needles, avoiding needles, hard to handle/ manipulate with hands, frequency of injections and difficult to handle during travel)	218	71%
No specific reason	16	5.3%
Infusion administration		
Reasons for choosing injection route:	22	7.2%
Feeling comfortable with someone else administrating the medication	3	1%
Multiple reasons (Feeling comfortable with someone else administrating the medication, frequency of medication doses, feeling of safety)	19	6.2%
Reasons for avoiding injection route:		
Inconvenient	119	38.8%
Multiple reasons (Inconvenient, and long infusion time)	90	29.3%
No specific reason	76	24.7%

Association between patients’ demographics and mode of therapy

We found no significant differences between the preferred mode of therapy and different demographic variables, as shown in Table 4.

**Table 4:** Association between mode of therapy and patients’ demographics (n= 307)

Variables		Mode of therapy			p-value
		Oral	Injection	Infusion	
Gender	Male	39	11	1	0.283
	Female	187	49	21	
Marital Status	Married	168	48	17	0.554
	Single	28	4	4	
	Divorced	12	2	1	
	Widowed	17	6	0	
Type of Residence	City	198	54	19	0.875
	Village	27	6	3	
Place of Residence	Central Region	7	7	2	0.172
	Eastern Region	2	2	0	
	Northern Region	6	2	1	
	Southern Region	68	19	6	
	Western Region	142	30	13	
Educational Level	University Degree	107	34	11	0.251
	Primary School	41	11	5	
	High School	47	7	1	
	Literate/No Formal Education	2	2	0	
	None	28	6	5	
Employment Status	Employed	63	23	7	0.649
	Unemployed	138	32	13	
	Retired	24	5	2	
Smoking	Yes	28	6	1	0.501
	No	197	54	21	

## Discussion

Studies addressing patients’ perspectives on their care are limited in Saudi Arabia. This is a crucial issue in shared decision-making for rheumatologists and patients. The present national study among RA patients in Saudi Arabia demonstrates that the oral route was the most commonly preferred mode of therapy (73.3%), followed by injection (19.5%), and IV infusion (7.3%), as shown in Figure 1. Our patients cited various reasons behind their choices. Ease of administration, recall, and obtaining the medications greatly influenced their choices. Difficulties in remembering to take medications and in swallowing the drugs were among the reasons for avoiding the oral route and selecting other therapies. Our patients found the convenience of use and frequency of medication doses (long intervals) to be reasons for selecting the injection route. However, fear of needles and the pain caused by them were among the reasons favoring the oral route, as detailed in Table 2 and Table 3. Interestingly, the majority of our patients were living in urban areas, unemployed, married, and non-smokers, as shown in Table 1. None of these factors were correlated with the patients’ choice of therapy in our regression analysis, as illustrated in Table 4.

Oral administration as the preferred mode of RA treatment ranked first in our cohort, aligning with many other global studies. However, the preference percentage likely varies from one country to another [3]. A multinational study with a smaller sample size than our current study found that 57% of RA patients ranked oral therapy first, while 29% selected self-injection, 16% opted for infusion and 2% chose clinic injection [4]. Other studies from different parts of the world, conducted among various populations of RA patients, found that approximately 50% or more of RA patients preferred the oral mode of therapy (53.7% [5], 49.1% [6], 56.4% [7]). Notably, oral therapy was more favored by our patients than in these studies.

Clearly, multiple factors across different societies make such comparisons less reliable, but they should at least provide direction to local policymakers. Demographics and disease characteristics such as the age of onset, first joints involved, progression, autoantibodies profile, and drugs used for management are examples of these factors. Recently published systematic reviews about attributes of treatment preference studies among RA patients [8,9], and the at-risk population [9] addressed issues like cost, side effects, treatment efficacy, frequency of treatment administration, and mode of administration. The reviews did not focus on studies that addressed the mode of therapy based on patients’ choices for medications with assured efficacy and safety. Instead, they addressed a complex issue beyond the scope of our study. Overall, as stated in other studies [10], variabilities in patients’ preferences were well represented, highlighting the importance of a shared decision-making process between rheumatologists and their patients.

Patients in general are well aware of the different modes of therapy available for various diseases. Throughout their lifetime, most patients have tried nearly all types of interventions. Among our cohort of RA patients, the oral mode of administration stood out as the most preferred mode of therapy. The introduction of new effective oral therapies for RA, such as JAK inhibitors, may have influenced this preference. The ease of handling oral therapy was by far the most common reason for choosing it, as shown in Table 2. It’s important to note that we did not address adherence issues in our study. Previous research has demonstrated that switching from oral to subcutaneous methotrexate increased patients’ adherence from 42% to 50.7% and increased the proportion of patients with remission or low disease activity from 22.8% to 52.9% [11]. It has also been shown that newer modes of therapy have influenced patients’ choices [10]. For instance, newer auto-injections were preferred by RA patients over pre-filled syringes and currently marketed auto-injectors [10]. However, the majority of our patients (71%) reported major concerns with the use of needles, citing issues such as pain, fear of needles, difficulties handling injections with hands, frequency of injections, and usability during travel.

The prior experiences of patients with different drugs might have influenced the findings of our study. We chose not to include previous patients’ exposure in our analysis to avoid complicating our results. Another factor worth mentioning is that our RA population may generally have less severe disease compared to those in other parts of the world [12] and may present with milder rather than severe disease [1]. A survey among RA patients from the US published in an abstract format [13] found a greater preference for non-oral routes among patients with more severe (vs. less severe) disease and among current bDMARD (vs. previous/never bDMARD) patients. However, the timing of that survey in 2016 might not reflect the widespread current use of oral JAK inhibitors. It is not feasible to allow patients to try all modes of administration and then choose their most favored one, as was suggested in one study [4]. From our local registry [1] and other many reported registries [14-16], we know that almost 30% or more of patients may require additional therapies with b/tsDMARDs. This does not underestimate the value of including prior patient experiences in similar studies, especially those with larger sample sizes. In a discrete-choice study among 733 Canadian patients [17], those with no experience with injectable treatments had preferences for oral administration relative to infusion. On the other hand, those with experience with injectable treatment did not show any significant preferences for one treatment mode over another.

Limitations

There are notable limitations in this study. The results demonstrated here are country-specific and cannot be generalized to other societies. The patients included in our analysis were primarily from the western region of Saudi Arabia. We do not anticipate major differences if true representations of all regions in the country were included in our analysis since all patients live under the same healthcare system. We tried to minimize participation bias by including patients from different centers. Patients were called more than once by trained interviewers. It was challenging for us to study adherence with an objective approach. We did not include any information about the disease activity measures of our patients, and we did not assess prior patients’ exposure to different modes of therapy.

## Conclusions

Despite these limitations, our study answered an important question for policymakers in our country. The oral route is the most preferred mode of therapy among RA patients in Saudi Arabia, followed by SC injection and finally IV infusions. The ease of handling stood out as the most common reason for considering any mode of therapy. A general fear of needles was common in our cohort of RA patients. These findings should provide insight into treating rheumatologists when discussing treatment options with their patients and considering shared decision-making strategies.
